# Linking personality traits to psychological distress among early-career PhD faculty: a gender-based canonical analysis

**DOI:** 10.3389/fpsyg.2025.1661608

**Published:** 2025-12-03

**Authors:** Tao Zhang, Tanghui Li, Maoyuan Pan, Shurong Jia, Qiang Wang, Hailin Ma, Hao Li

**Affiliations:** 1 Mental Health Education Center, Xihua University, Chengdu, China; 2 School of Physical Science and Engineering, Beijing Jiaotong University, Beijing, China; 3 Tibet Autonomous Region Key Laboratory for High Altitude Brain Science and Environmental Acclimatization, Tibet University, Lhasa, China; 4 Plateau Brain Science Research Center, Tibet University, Lhasa, China

**Keywords:** young doctoral faculty, personality trait, mental health, gender differences, canonical correlation analysis

## Abstract

**Objective:**

Early-career doctoral faculty face unique stressors that may impact their mental health, yet gender-specific personality-mental health patterns in this population remain underexplored, especially within Chinese higher education.

**Methods:**

Using simple random sampling, 329 newly appointed doctoral faculty members (<35 years, <1 year teaching experience) were recruited from three comprehensive universities in Chengdu, Southwest China. Personality traits were measured with the Sixteen Personality Factor Questionnaire (16PF), and mental health was assessed using the Symptom Checklist-90 (SCL-90). Descriptive, comparative, and canonical correlation analyses (CCA) were conducted, with interpretation based on effect sizes (Rc^2^) and structure coefficients (|rs| ≥ 0.30) rather than nominal *p*-values.

**Results:**

About 16.7% of participants screened positive for psychological distress, with obsessive-compulsive symptoms and interpersonal sensitivity being most frequent. Female faculty reported higher anxiety than males. Gender differences also emerged in personality profiles, with men scoring higher in dominance, privateness, and self-reliance, and women higher in warmth and abstractedness. CCA revealed moderate, theoretically consistent associations between personality and mental-health dimensions (men: Rc = 0.57, Rc^2^ = 32%; women: Rc = 0.41, Rc^2^ = 16.9%). Social boldness and rule-consciousness were inversely linked to interpersonal and somatic distress, whereas tension and apprehension predicted broader symptom elevation.

**Conclusion:**

Personality configurations are moderately associated with mental-health outcomes among early-career doctoral faculty, with stronger multivariate coupling in men. Findings highlight the need for gender-sensitive, person–environment-fit interventions—such as supportive mentoring, balanced workloads, and accessible counseling—to promote sustainable well-being in academic settings.

## 1 Introduction

With the continuous development of higher education, young doctoral faculty members have gradually become an important part of the academic system in universities. However, as representatives of academic talent, these young faculty members are not only required to undertake heavy teaching responsibilities but also face challenges related to research pressures, career development, and personal life (Brailovskaia et al., 2024). These challenges often have profound effects on their mental health and professional growth, which in turn directly influence the quality of education they deliver. Therefore, studying the relationship between personality traits and mental health, particularly within the context of young doctoral faculty members in higher education, holds considerable theoretical and practical value.

Mental health has been recognized as one of the key factors in professional development, and personality traits may play an important role in shaping faculty members’ psychological well-being (Ren and Wang, 2022). Personality traits significantly influence individuals’ ability to cope with stress, handle professional setbacks, and regulate their emotions (Pei et al., 2022). In the field of higher education, faculty members in universities represent a new force in academia, shouldering multiple roles including teaching, research, and social service (Gehrke and Kezar, 2015). Their mental health and personality traits are not only critical to their individual career development and quality of life but also have a profound impact on the quality of education and the academic atmosphere in universities. From an educational perspective, supporting the psychological well-being of these faculty members is essential for sustaining a high-quality, student-centered teaching environment. With the intensification of social competition and increasing work pressure, the mental health issues of young doctoral faculty members have gradually received more attention (Byrom et al., 2022; Casey et al., 2023). In parallel, accumulating evidence links personality—an anchor of individual differences—to mental-health outcomes in occupational settings (Kang et al., 2023). Clarifying these links in the context of early academic careers can advance psychological theory and provide a data-informed basis for faculty development programs, targeted mental-health services, and work-design policies that foster sustainable, student-centered higher education.

Existing research has shown that faculty members’ mental health directly impacts their teaching effectiveness, job satisfaction, and career development (Capone and Petrillo, 2020; Singh and Gautam, 2024). Although existing research has begun to focus on the mental health issues of university faculty members, studies specifically targeting a distinct group—newly appointed young doctoral faculty members—are relatively limited (Hammoudi Halat et al., 2023). As a key region in China’s higher education system, the Southwestern region has seen insufficient research on the mental health and personality traits of young doctoral faculty members in its universities. Most existing research tends to concentrate on single-dimensional aspects, such as anxiety, depression, or occupational stress, lacking a systematic exploration of the multidimensional relationship between personality traits and mental health (Ebstrup et al., 2011; Klein et al., 2011; Nouri et al., 2019). This study aims to address the existing research gap by using multivariate statistical methods to comprehensively analyze the mental health status and personality traits of young doctoral faculty members in universities in Southwest China, while also exploring the interrelationship between these two factors. Importantly, this research provides evidence-based insights that can inform educational policy and institutional practices aimed at fostering healthier, more resilient faculty populations.

Additionally, gender plays a pivotal yet understudied role in mental health. While men and women face similar academic pressures, their psychological responses differ significantly. Female faculty members report higher anxiety levels due to societal expectations about caregiving roles and workplace gender biases (McLean and Anderson, 2009). Work–family conflict is particularly pronounced for women, as there is an asymmetry in the division of work and family roles between women and men (McElwain et al., 2005). These gendered dynamics create distinct mental health risks that require targeted interventions. Addressing such differences can help institutions design more equitable and effective support systems, thereby contributing to a more inclusive and psychologically supportive educational environment.

This study aims to examine the multidimensional relationship between personality traits and mental health among young doctoral faculty members in Chinese higher-education institutions. Using the Sixteen Personality Factor Questionnaire (16PF) (Cattell and Mead, 2008) and the Symptom Checklist-90 (SCL-90) (Derogatis and Unger, 2010), we assessed how specific personality traits relate to psychological symptom dimensions and whether these associations differ by gender. By applying canonical correlation analysis (CCA), we sought to move beyond single-variable comparisons and identify integrated patterns linking personality configurations to mental-health outcomes.

Based on prior empirical and theoretical findings, we formulated the following hypotheses:

(1) Compared with the Chinese national adult norms, young doctoral faculty members would demonstrate better overall mental health profiles on the SCL-90, reflecting adaptive adjustment despite occupational stressors.
(2) Female doctoral faculty members would report higher anxiety levels than male counterparts, partly attributable to gender-linked personality patterns such as heightened sensitivity or tension.
(3) Personality traits and mental-health dimensions would exhibit significant multivariate associations, such that specific personality factors (e.g., boldness, emotional stability, tension) are systematically related to mental-health outcomes across genders.

## 2 Methods

### 2.1 Participants

A simple random sampling approach was used to recruit young doctoral faculty members from three comprehensive universities in Chengdu, Southwest China. The three universities were selected because they have comparable educational quality, faculty recruitment criteria, and teaching loads. Within each institution, we obtained lists of newly appointed doctoral faculty members (aged under 35 years, <1 year of teaching experience) from the Human Resources Offices and randomly selected potential participants using computer-generated numbers. This study was reviewed and approved by Xihua University Research Ethical Board. Written informed consent for participation in the study was provided by the participants.

A total of 460 invitations were distributed (approximately 150–160 per university), and 329 valid responses were collected (response rate = 71.5%). Responses with extremely short or long completion times and those from individuals aged >35 were excluded to ensure data quality and representativeness of newly hired faculty. The final sample included 152 male (46.2%) and 177 female (53.8%) doctoral faculty members, aged 27–35 years. Participation was voluntary and anonymous, and all participants provided informed consent.

### 2.2 Measures

#### 2.2.1 Mental health

The SCL-90 was developed by L. R. Derogatis in 1975 and consists of 90 items, which assess psychological health across 10 dimensions: somatization, obsessive-compulsive symptoms, interpersonal sensitivity, depression, anxiety, hostility, phobic anxiety, paranoid ideation, psychoticism, and others (including sleep and eating disturbances) (Dang et al., 2021). The scale reflects the psychological health status of the respondent over the past week, with the results being time-sensitive. Responses are scored on a 5-point Likert scale, ranging from 1 to 5 according to the severity of symptoms, with higher scores indicating more severe psychological distress. The total score is the sum of the individual item scores.

#### 2.2.2 Personality traits

The Sixteen Personality Factor Questionnaire (16PF) was originally developed by Raymond B. Cattell in the 1940s and 1950s, with the most widely used Fifth Edition published by Cattell and colleagues in 1993 (Cattell and Mead, 2008). The scale categorizes human inherent and stable traits into 16 factors: warmth, reasoning, emotional stability, dominance, liveliness, rule-consciousness, social boldness, sensitivity, vigilance, abstractedness, privateness, apprehension, openness to change, self-reliance, perfectionism, and tension. These factors are psychometrically independent of each other. The questionnaire consists of 187 items, with most items scored on a 3-point scale (0, 1, 2 or 2, 1, 0). Only the reasoning item has a single correct answer, scored dichotomously (1 point for correct, 0 for incorrect).

### 2.3 Canonical correlation analysis

To examine the multivariate association between personality traits and mental-health dimensions, we performed canonical correlation analysis (CCA) using the 16PF primary factors (16 variables) as predictors and the SCL-90 symptom dimensions (9 variables) as criteria (Hardoon et al., 2004). All variables were standardized (z-scores) prior to analysis. The CCA was performed separately for men and women, as well as for the total sample, to explore potential gender differences in the multivariate association patterns (Akour et al., 2023; Li et al., 2023). Although both scales include a large number of items, the analysis was conducted at the dimension level (16 personality factors and 9 symptom dimensions) rather than at the item level. With 25 composite variables and 329 participants, the sample size was sufficient according to established guidelines for canonical correlation analysis (Stevens, 2002).

Before performing the CCA, statistical assumptions were verified. Multicollinearity within each variable set was examined using variance inflation factors (VIFs) (Alves et al., 2017). All VIFs were below the recommended threshold of 5 (Men: 16PF max VIF = 2.60; SCL-90 = 4.13; Women: 16PF = 2.48; SCL-90 = 3.87), suggesting acceptable collinearity. Linearity and multivariate normality were evaluated via scatterplot matrices and Q–Q plots of the canonical variates, revealing approximately linear relationships. These deviations were deemed acceptable for linear CCA.

Canonical functions were extracted sequentially, each representing a pair of synthetic variates (u, v) that maximize the shared variance between the 16PF and SCL-90 variable sets. The significance of each canonical function was assessed using Wilks’ *λ* with Bartlett’s χ^2^ approximation. Because the CCA was conducted across three subsamples (total, men, and women) and multiple canonical roots, we applied the Benjamini–Hochberg false discovery rate (FDR) correction (Benjamini and Hochberg, 1995) to control for inflated Type I error due to multiple testing. After adjustment, none of the canonical functions reached *q* < 0.05, and results were thus interpreted as exploratory and descriptive rather than inferential.

Following established guidelines (Sherry and Henson, 2005; Thompson, 1984), two complementary criteria were used to determine which canonical functions were retained for interpretation: (1) Only canonical functions with Rc ≥ 0.30 (i.e., explaining ≥ 9% shared variance, Rc^2^ ≥ 0.09) were considered substantively meaningful. (2) Retained canonical functions were further examined for variables with salient structure coefficients (|rs| ≥ 0.30) on either the 16PF or SCL-90 side, indicating meaningful contribution to the canonical variate. Functions not meeting both criteria were treated as statistically or substantively negligible and were reported for completeness but not interpreted. This approach emphasizes effect size and structural interpretability over nominal statistical significance, aligning with modern recommendations for multivariate psychological research (Hair, 2009).

### 2.4 Correlation analysis

Pearson correlation analysis, based on univariate methods, was used to examine the correlations between the 9 factors of the SCL-90 scale and the 16 dimensions of the 16PF scale. A correlation was considered significant when *p* < 0.05. Finally, to further explore the relationship between personality traits and mental health, CCA was conducted to examine the relationship between the 16 dimensions of the 16PF and the 9 factors of the SCL-90.

### 2.5 Statistical analysis

Data analysis was conducted using SPSS 23.0 software.^1^ Continuous variables were first assessed for normality, and descriptive statistics were reported as “mean ± standard deviation.” A one-sample *t*-test (two-tailed) was used to compare the mean scores of the total sample on the SCL-90 and 16PF scales with the national norm. An independent samples *t*-test (two-tailed) was employed to compare the SCL-90 and 16PF scale scores between male and female doctoral faculty groups. Statistical significance was set at *p* < 0.05.

## 3 Results

### 3.1 Bivariate correlation analysis between personality traits and mental health

When comparing the mental health of young doctoral faculty members with the national norm, we identified some noteworthy results. The results of the bivariate correlation analysis between personality traits and mental health are presented in Table 1. Based on the SCL-90 scale, mental health was assessed using a threshold of a total score exceeding 160 points or a mean score greater than 2 for any factor (Fang et al., 2023; Hou et al., 2020). The results showed that the prevalence of mental health issues among young doctoral faculty members was 16.70% (55/329). Among the SCL-90 factors, the prevalence rates from highest to lowest were: obsessive-compulsive symptoms 11.90% (39/329), interpersonal sensitivity 6.08% (20/329), other symptoms (sleep and eating disturbances) 4.56% (15/329), and paranoid ideation 2.74% (9/329). These data suggest that young doctoral faculty members face significant mental health challenges, particularly regarding obsessive-compulsive symptoms and interpersonal sensitivity.

**Table 1:** Bivariate correlation analysis between 16PF and SCL-90.

	SOM	OC	IS	DEP	ANX	HOS	PHOB	PAR	PSY
Warmth A	−0.01	0.05	−0.05	−0.01	0.03	−0.05	−0.07	−0.00	−0.02
Reasoning B	0.06	−0.05	−0.10	−0.03	−0.04	−0.04	−0.05	−0.03	−0.05
Emotional Stability C	0.03	−0.02	−0.09	−0.10	−0.07	−0.09	**−0.14** ^**^	−0.02	−0.09
Dominance E	0.02	−0.03	−0.06	−0.07	−0.04	−0.02	−0.10	0.05	−0.03
Liveliness F	−0.06	−0.05	−0.08	**−0.14** ^*^	−0.06	−0.06	−0.09	−0.03	−0.08
Rule-Consciousness G	0.03	−0.04	−0.10	−0.08	−0.10	−0.09	**−0.15** ^**^	−0.08	−0.05
Social Boldness H	0.01	−0.09	**−0.14** ^*^	**−0.14** ^*^	−0.07	−0.09	**−0.15** ^**^	−0.02	−0.07
Sensitivity I	−0.03	0.04	0.01	0.05	0.02	0.10	0.04	0.02	0.08
Vigilance L	−0.01	0.00	0.05	0.03	0.03	0.04	0.11	0.00	0.02
Abstractedness M	0.00	−0.03	−0.05	−0.05	0.01	0.02	−0.01	−0.03	−0.02
Privateness N	−0.03	−0.06	0.01	0.05	0.01	0.02	−0.05	0.09	−0.03
Apprehension O	0.06	0.06	**0.15** ^**^	**0.20** ^***^	**0.12** ^*^	**0.14** ^*^	**0.16** ^**^	0.06	0.08
Openness to Change Q1	0.06	−0.01	−0.01	−0.02	0.00	0.03	−0.04	0.01	0.06
Self-Reliance Q2	**0.12** ^*^	0.05	0.05	0.05	0.04	0.05	−0.02	0.10	0.07
Perfectionism Q3	0.03	−0.01	−0.07	−0.08	−0.01	0.01	−0.06	0.01	−0.03
Tension Q4	0.06	0.07	**0.15** ^**^	**0.19** ^**^	**0.11** ^*^	**0.14** ^*^	**0.20** ^***^	0.08	**0.17** ^**^

SOM, somatization; OC, obsessive-compulsive; IS, interpersonal sensitivity; DEP, depression; ANX, anxiety; HOS, hostility; PHOB, phobic anxiety; PAR, paranoid ideation; PSY, psychoticism; *p ≤ 0.05, **p ≤ 0.01, ***p ≤ 0.001. Bold text indicates a significant correlation.

When comparing the 16PF scores with the national norm, doctoral faculty members scored significantly higher than the national norm in reasoning, emotional stability, dominance, liveliness, rule-consciousness, social boldness, privateness, and perfectionism (*p* < 0.01). Conversely, they scored significantly lower in sensitivity, vigilance, apprehension, openness to change, and tension (*p* < 0.01).

Pearson correlation analysis revealed significant associations between the 16PF and the SCL-90 subscales. Three traits—social boldness, apprehension, and tension—emerged as key predictors of mental health outcomes. Specifically, social boldness demonstrated significant negative correlations with interpersonal sensitivity (*r* = −0.14), depression (*r* = −0.14), and phobic anxiety (*r* = −0.15), suggesting that boldness may enhance psychological resilience. Apprehension was positively associated with interpersonal sensitivity (*r* = 0.15), anxiety (*r* = 0.12), and hostility (*r* = 0.14), with particularly strong correlations observed for phobic anxiety (*r* = 0.16, *p* < 0.01) and depression (*r* = 0.20, *p* < 0.01). Similarly, tension correlated positively with phobic anxiety (*r* = 0.20), depression (*r* = 0.19), psychoticism (*r* = 0.17), interpersonal sensitivity (*r* = 0.15), hostility (*r* = 0.14), and anxiety (*r* = 0.11), with the strongest associations observed for depression and phobic anxiety (*p* < 0.01). These findings indicate that higher apprehension and tension are associated with poorer mental health outcomes, whereas greater boldness may correlate with better mental health indicators.

### 3.2 Gender differences in mental health status

Analysis of gender differences in mental health among young doctoral faculty members showed minimal sex-based variation. As shown in Table 2, mental health outcomes were significantly correlated with gender. Anxiety was the only subscale demonstrating a significant sex difference, with female participants scoring higher than males (*t*_2_ = −2.69, *p* < 0.01). No significant differences emerged across other SCL-90 subscales: somatization, obsessive-compulsive, interpersonal sensitivity, depression, hostility, phobic anxiety, paranoid ideation, or psychoticism (*p* > 0.05).

**Table 2:** Comparison of mental health status among new young doctoral faculty members.

Variables	Group		*t*_1_	Gender		*t*_2_
	National model (*N* = 1,388)	Sample (*n* = 329)		Male (*n* = 152)	Female (*n* = 177)	
Total	1.44 ± 0.43	1.29 ± 0.21	**−13.24** ^***^	1.28 ± 0.22	1.30 ± 0.19	−1.20
SOM	1.37 ± 0.48	1.17 ± 0.21	**−16.87** ^***^	1.16 ± 0.21	1.19 ± 0.21	−1.33
OC	1.62 ± 0.58	1.58 ± 0.31	−2.32	1.55 ± 0.35	1.60 ± 0.28	−1.50
IS	1.65 ± 0.51	1.36 ± 0.31	**−17.01** ^***^	1.35 ± 0.34	1.36 ± 0.28	−0.22
DEP	1.50 ± 0.59	1.24 ± 0.24	**−19.93** ^***^	1.22 ± 0.24	1.26 ± 0.24	−1.39
ANX	1.39 ± 0.43	1.30 ± 0.23	**−6.68** ^***^	1.27 ± 0.24	1.34 ± 0.23	**−2.69** ^**^
HOS	1.46 ± 0.56	1.21 ± 0.23	**−19.16** ^***^	1.19 ± 0.24	1.23 ± 0.23	−1.36
PHOB	1.23 ± 0.41	1.11 ± 0.18	**−11.50** ^***^	1.20 ± 0.17	1.13 ± 0.19	−1.78
PAR	1.43 ± 0.57	1.28 ± 0.27	**−10.17** ^***^	1.29 ± 0.30	1.27 ± 0.25	0.63
PSY	1.29 ± 0.42	1.26 ± 0.24	−2.59	1.24 ± 0.25	1.27 ± 0.23	−0.81

SOM, somatization; OC, obsessive-compulsive; IS, interpersonal sensitivity; DEP, depression; ANX, anxiety; HOS, hostility; PHOB, phobic anxiety; PAR, paranoid ideation; PSY, psychoticism; t_1_ represents the comparison of the SCL-90 average scores between the total sample of new young doctoral faculty members and the national norm was tested using a one-sample *t*-test. t_2_ statistic represents the difference in SCL-90 average scores between male and female doctoral faculty members, tested using a two-sample *t*-test, **p < 0.01, ***p < 0.001. Bold text indicates significant differences.

### 3.3 Gender differences in personality traits

The comparison revealed significant gender differences in specific personality traits among young doctoral faculty members. The results of the gender differences in personality traits are presented in Table 3. Male participants scored significantly higher than females in dominance (*t*_3_ = 2.63, *p* < 0.01), privateness (*t*_3_ = 2.71, *p* < 0.01), and self-reliance (*t*_3_ = 2.65, *p* < 0.01). Conversely, females exhibited significantly greater warmth (*t*_3_ = −2.82, *p* < 0.01) and abstractedness (*t*_3_ = −2.92, *p* < 0.01). No significant gender differences were observed in reasoning, emotional stability, liveliness, rule-consciousness, social boldness, vigilance, apprehension, openness to change, perfectionism, or tension. Both genders exhibited comparable deviations from the national norm, including elevated scores in emotional stability, dominance, liveliness, rule-consciousness, social boldness, and perfectionism, alongside reduced scores in sensitivity, vigilance, apprehension, openness to change, self-reliance, and tension. The most marked contrasts emerged in interpersonal dimensions, with females showing stronger affiliative tendencies in warmth and males displaying greater assertiveness in dominance and independence in self-reliance, reflecting divergent interpersonal orientations between genders.

**Table 3:** Comparison of personality traits among new young doctoral faculty members.

16PF	National model		Young doctoral faculty members				*t*_3_
	Male (N = 830)	Female (N = 805)	Male (n = 152)	*t*_1_	Female (n = 177)	*t*_2_	
Warmth A	9.63 ± 3.17	10.10 ± 3.29	10.22 ± 3.33	2.18	11.39 ± 4.10	4.19***	−2.82**
Reasoning B	7.98 ± 2.05	8.69 ± 1.94	9.21 ± 1.29	11.76***	9.01 ± 1.41	3.04**	1.33
Emotional Stability C	15.16 ± 3.27	14.76 ± 3.39	19.84 ± 3.29	17.52***	18.92 ± 3.28	16.88***	2.54
Dominance E	11.18 ± 3.56	9.92 ± 3.12	13.26 ± 2.99	8.60***	12.39 ± 3.01	10.92***	2.63**
Liveliness F	10.74 ± 3.77	10.42 ± 3.86	16.48 ± 3.87	18.31***	16.62 ± 3.63	22.73***	−0.33
Rule-Consciousness G	13.25 ± 3.23	13.91 ± 3.16	15.91 ± 2.23	14.74***	15.59 ± 2.48	9.03***	1.23
Social Boldness H	9.95 ± 3.71	8.85 ± 3.58	15.24 ± 3.96	16.48***	15.59 ± 4.04	21.71***	−0.47
Sensitivity I	11.11 ± 2.80	11.81 ± 2.58	9.34 ± 3.36	−6.50***	10.30 ± 3.70	−5.43***	−2.44
Vigilance L	11.12 ± 2.39	10.82 ± 2.68	5.43 ± 2.79	−25.10***	5.51 ± 2.98	−23.73***	−0.23
Abstractedness M	12.62 ± 3.25	12.97 ± 3.39	12.68 ± 2.51	0.32	13.56 ± 2.87	2.73**	−2.92**
Privateness N	8.71 ± 2.56	9.25 ± 2.45	10.70 ± 2.07	11.81***	10.06 ± 2.15	5.02***	2.71**
Apprehension O	11.03 ± 3.54	12.35 ± 3.42	6.60 ± 2.67	−20.44***	6.73 ± 3.08	−24.27***	−0.41
Openness to Change Q1	11.78 ± 2.92	11.27 ± 2.97	10.25 ± 1.95	−9.65***	10.21 ± 2.23	−6.34***	0.18
Self-Reliance Q2	12.75 ± 3.30	11.98 ± 3.15	11.40 ± 2.61	−6.36***	10.59 ± 2.88	−6.41***	2.65**
Perfectionism Q3	12.70 ± 3.04	13.05 ± 2.94	15.45 ± 2.08	16.32***	15.73 ± 2.10	16.96***	−1.19
Tension Q4	10.83 ± 3.60	11.70 ± 3.97	6.49 ± 2.98	−18.00***	6.45 ± 3.16	−22.13***	0.12

SOM, somatization; OC, obsessive-compulsive; IS, interpersonal sensitivity; DEP, depression; ANX, anxiety; HOS, hostility; PHOB, phobic anxiety; PAR, paranoid ideation; PSY, psychoticism; t_1_ represents the difference in scores between male doctoral faculty members and the national male norm; t_2_ represents the difference in scores between female doctoral faculty members and the female national norm; and t_3_ represents the difference in 16PF average scores between male and female doctoral faculty members; **p < 0.01, ***p < 0.001. Bold text indicates significant differences.

### 3.4 Canonical correlation analysis between personality traits and mental health

To further explore the overall relationship between personality traits and mental health, we conducted a canonical correlation analysis using the 16PF scores as predictor variables and SCL-90 factors as criterion variables. This analysis aimed to extract the canonical relationships between personality traits and mental health factors. Table 4 shows canonical correlations, effect sizes, and confidence intervals for the first two canonical functions across groups. Although none of the Wilks’ *λ* tests reached statistical significance (all *p* > 0.05), the first canonical correlations in both men and women exceeded the *a priori* threshold of Rc ≥ 0.30, with moderate effect sizes (Rc^2^ ≈ 14–32%) and 95% confidence intervals not including zero. These patterns indicate that, despite limited statistical power under high dimensionality, the associations between the 16PF and SCL-90 variable sets were meaningful and consistent with theoretical expectations. Therefore, interpretation focused on effect sizes and structure coefficient patterns rather than on nominal significance levels.

**Table 4:** Canonical correlation results between 16PF and SCL-90 by gender.

Canonical function	Rc	Rc^2^ (%)	Wilks’ λ	df	*p*-value	Rc 95% CI	Interpretation
Total (*N* = 329)							
U1: V1	0.37	14.0	0.65	144	0.65	[0.28, 0.46]	Moderate effect
U2: V2	0.32	10.2	0.75	120	0.98	[0.22, 0.41]	Small effect
Male (*n* = 152)							
U1: V1	0.57	32.0	0.30	144	0.12	[0.45, 0.67]	Moderate-to-large effect
U2: V2	0.46	21.2	0.45	144	0.98	[0.33, 0.58]	Small-to-moderate effect
Female (*n* = 177)							
U1: V1	0.41	16.9	0.40	144	0.33	[0.28, 0.53]	Moderate effect
U2: V2	0.40	15.9	0.48	144	0.92	[0.27, 0.52]	Moderate effect

Rc = canonical correlation coefficient; Rc^2^ = shared variance (effect size) between variable sets; Wilks’ λ = sequential canonical function test (Bartlett χ^2^ approximation). 95% confidence intervals were derived using Fisher’s z transformation. Interpretation is based on effect size magnitude rather than statistical significance, given the high dimensionality (16 × 9 variables) and moderate sample sizes.

**Figure 1:**
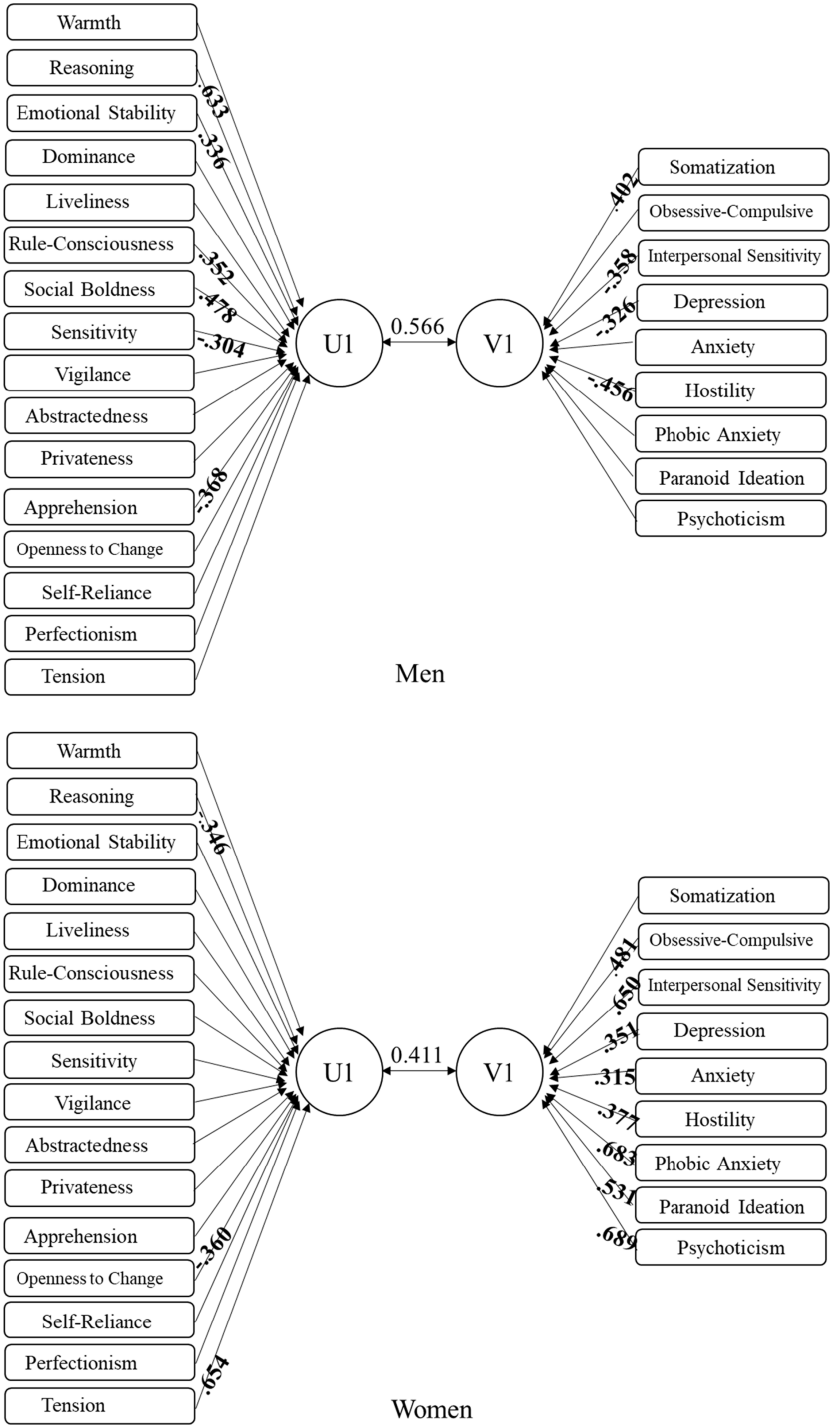
Structure coefficient of the first canonical factors in men and women.

To further characterize the multivariate association between personality traits and mental-health symptoms, we visualized structure coefficients (rs) for the first canonical function in men and women (Figure 1), alongside the canonical effect sizes and uncertainty estimates summarized in Table 4. In men (Rc = 0.57; Rc^2^ = 32%), salient loadings (|rs| ≥ 0.30) were observed for Social Boldness (H), Reasoning (B), and Rule-Consciousness (G), coupled with higher loadings on Interpersonal Sensitivity (IS), Hostility (HOS), and Somatization (SOM) on the SCL-90 side, delineating a socially confident and cognitively engaged profile inversely associated with interpersonal-emotional distress and bodily complaints. In women (Rc = 0.41; Rc^2^ = 16.9%), the first root was characterized by higher loadings for Tension (Q4) and Openness to Change (Q1), covarying with Phobic Anxiety (PHOB), Psychoticism (PSY), and Depression (DEP), suggesting that heightened arousal/reactivity co-occurs with broader symptom elevation. Although sequential Wilks’ tests did not survive FDR correction (all *q* > 0.05), the canonical correlations and their confidence intervals (Table 4) indicate moderate, interpretable covariation patterns that are consistent with theoretical expectations. We therefore emphasize effect sizes (Rc^2^) and structure-coefficient patterns (Figure 1) rather than nominal *p*-values, given the high dimensionality (16 × 9) and moderate sample sizes.

## 4 Discussion

This study examined how personality traits relate to mental-health symptoms among young doctoral faculty in a Chinese university context. Across bivariate and multivariate lenses, the picture that emerges is consistent and theoretically coherent: personality traits associated with social approach and adaptive control (e.g., social boldness, openness to change, emotional stability, rule-consciousness) tend to co-occur with lower interpersonal and somatic distress, whereas personality traits reflecting internal tension and apprehension tend to co-occur with broader symptom elevation. These links were moderate in magnitude and should be understood as covariation rather than prediction or causation.

### 4.1 Integrative interpretation of personality–mental-health linkages

Bivariate analyses revealed that obsessive-compulsive symptoms and interpersonal sensitivity were the most prevalent concerns (≈17%), confirming that mental-health risks are non-trivial even among high-achieving early-career faculty. These findings are consistent with previous research (Pengju et al., 2018; Zhang et al., 2022). Although young doctoral faculty members exhibit certain mental health concerns, their average scores for somatization, interpersonal sensitivity, depression, anxiety, hostility, phobic anxiety, and paranoid ideation were all lower than the national norm, indicating a relatively better mental health status compared to the general population (Poropat, 2009). When compared with national (Chinese) norms, participants showed a profile typical of academically selected groups—higher reasoning, emotional stability, dominance, liveliness, rule-consciousness, social boldness, privateness, and perfectionism but lower sensitivity, vigilance, apprehension, openness to change, and tension—suggesting generally good mental health but uneven adaptation across domains.

CCA integrated these findings at the multivariate level. The first canonical function captured a continuum between agentic personality characteristics and interpersonal–somatic complaints, consistent with person–environment fit and stress-coping models (Caplan, 1987; Kristof-Brown et al., 2005). Faculty who displayed greater social courage and task focus tended to report fewer interpersonal sensitivity and somatization symptoms, whereas those higher in tension and apprehension reported broader distress. These results portray personality traits as psychological filters shaping stress appraisal and coping in academic life.

### 4.2 Effect sizes and interpretive focus of the CCA analysis

The present study identified moderate canonical correlations linking personality traits and mental health dimensions among young doctoral faculty. Although the first canonical functions in both genders exhibited interpretable covariation patterns, the proportion of shared variance (Rc^2^ ≈ 14–32%) indicates that these associations are moderate in magnitude and explanatory scope. Canonical correlation analysis quantifies shared multivariate structure, not prediction or causation; therefore, the findings should be understood as descriptive evidence of how personality and mental-health variables co-vary, rather than as deterministic or predictive models.

The canonical dimensions reflect patterns of covariance that are statistically stable but account for only a modest proportion of the total variance, which is typical in multivariate psychological research (Sherry and Henson, 2005; Thompson, 1984). These results highlight that personality–mental health linkages are complex, multidimensional, and influenced by other contextual and situational factors not captured in the present analysis. Consequently, our interpretations focus on the direction and psychological coherence of the observed associations rather than on their magnitude as indicators of predictive accuracy.

### 4.3 Gender-related patterns and sociocultural mechanisms

The stronger canonical associations observed among men (Rc = 0.57, Rc^2^ = 32%) compared to women (Rc = 0.41, Rc^2^ = 16.9%) warrant further interpretation. This gender difference may reflect sociocultural and psychological factors that shape the expression of personality and mental health among early-career academics in China (McLean and Anderson, 2009).

First, gender role socialization may contribute to this pattern (Calsy and D’Agostino, 2021; Eagly and Karau, 2002; Galsanjigmed and Sekiguchi, 2023). In Chinese culture, male academics often face stronger achievement and status expectations, with greater pressure to demonstrate competence, rationality, and control (Horta and Tang, 2023). Such expectations may amplify the covariation between agentic personality traits (e.g., Social Boldness, Reasoning, Rule-Consciousness) and stress-related symptoms, as the tension between self-demands and role obligations manifests psychologically. In contrast, female academics may experience more diffuse role expectations—balancing professional and interpersonal domains—which could distribute psychological stress across multiple life areas, weakening the linear covariation between specific personality dimensions and symptom patterns. This finding aligns with broader societal pressures: women in academia often face dual burdens of professional excellence and caregiving expectations, which amplify stress and overthinking (Telayneh, 2019).

Second, gender differences in emotional expression and coping may also play a role. Prior studies have found that women tend to exhibit greater emotional awareness and social support-seeking, which may buffer the direct link between personality traits and psychopathological symptoms (Matud, 2004; Nolen-Hoeksema, 2012). Men, in contrast, are often socialized to maintain emotional restraint, leading to an accumulation of internalized tension that may intensify the personality–symptom association.

Third, potential reporting biases inherent in self-report measures should be acknowledged. Men may underreport emotional distress due to stigma or perceived social desirability, which can distort the covariance structure by attenuating symptom variability in milder ranges. Thus, the stronger canonical correlation in men should be interpreted not as greater vulnerability but as greater linear coherence between dispositional and symptomatic patterns.

Collectively, these interpretations suggest that gendered socialization processes, cultural norms, and measurement factors jointly shape how personality traits relate to mental health in early academic careers. The same personality traits influenced the mental health factors and their impact differently across genders, likely due to differing social roles and cognitive patterns between men and women (Eagly and Wood, 2013; Hyde, 2014). This highlights the importance of tailoring talent development strategies for young doctoral faculty members based on gender-specific needs (Ma et al., 2024).

### 4.4 Cross-cultural context and global comparison

The present findings align with a growing international body of research documenting the mental health challenges faced by early-career academics. Across regions, young faculty and doctoral researchers report high levels of psychological distress related to job precarity, publication pressure, and performance evaluation systems (Evans et al., 2018; Levecque et al., 2017). In the United Kingdom, Australia, and North America, early-career scholars experience similar conflicts between professional ideals and structural constraints, leading to anxiety, burnout, and decreased well-being (Oliveira et al., 2025). These studies echo our results showing that personality traits such as tension, boldness, and rule-consciousness are strongly linked with emotional and somatic stress among young academics.

However, the Chinese context introduces distinct sociocultural and institutional dynamics that may intensify these associations. In collectivist cultures, academic success is often viewed not merely as individual achievement but as a familial and institutional responsibility, which may heighten the psychological burden of failure or underperformance (Sun and Ryder, 2016). Moreover, the rapid expansion of higher education and the “publish-or-perish” evaluation system in China have increased competition, job insecurity, and administrative workload (Xu et al., 2023). These pressures may strengthen the covariance between agentic personality traits and stress-related symptoms, as individuals with strong achievement drives experience greater tension when facing constrained autonomy or limited institutional support.

In contrast to Western systems that often emphasize individual career agency and self-care practices, Chinese early-career academics operate within hierarchical mentorship structures and collectivist expectations, where emotional restraint and perseverance (“chiku”) are culturally valorized (Sze and Bao, 2025; Yuan and Tian, 2023). This cultural context may explain why personality–symptom linkages observed in our study—particularly among male faculty—reflect not only individual dispositions but also internalized cultural scripts about endurance, conformity, and self-regulation.

### 4.5 Implications for educational psychology and faculty support

From the lens of educational psychology, these results emphasize the role of person–environment fit and stress–coping alignment during the early academic career stage. Traits such as openness to change and social boldness appear adaptive under academic uncertainty, while high tension and perfectionism may heighten vulnerability. These findings align with person–environment congruence theory and stress-adaptation models (Oehlhorn et al., 2025; Salimzadeh et al., 2021), suggesting that mental health outcomes arise from the interplay between dispositional tendencies and institutional climates.

To translate these insights into practice, universities should implement early identification and support mechanisms for new faculty. Possible strategies include structured mentorship programs, regular mental-health assessments, stress-management workshops, and organizational reforms to balance administrative and research demands. Embedding psychological support into faculty development not only benefits individual well-being but also enhances organizational productivity and retention, contributing to a psychologically sustainable academic ecosystem.

### 4.6 Limitations

Several limitations should be acknowledged when interpreting the present findings. First, the study employed a cross-sectional design, which restricts any inference of causality. Although meaningful associations between personality traits and mental-health dimensions were identified, it remains uncertain whether personality predispositions influence subsequent psychological adjustment or whether early work-related stressors and adaptation processes shape the expression of personality traits. Longitudinal follow-up of early-career faculty would be essential to clarify these temporal dynamics and potential bidirectional effects.

Second, participants were drawn from three universities in Southwest China. Although these institutions share recruitment and evaluation systems consistent with national higher-education standards, regional cultural characteristics—such as stronger collectivist values and relatively moderate work competition—might influence interpersonal dynamics and self-perceived psychological stress. Therefore, the generalizability of the findings should be interpreted cautiously. Future research could replicate the study in other Chinese regions or different academic systems to assess cross-regional stability.

Third, both personality and mental-health variables were assessed through self-report instruments (16PF and SCL-90), which raises the possibility of response bias and common method variance. Although the survey was anonymous and included diverse item formats to reduce such bias, these procedures cannot fully eliminate it. Future research should combine multiple data sources—such as supervisor or peer evaluations, behavioral tasks, or physiological indicators—to strengthen the validity of the observed associations.

Fourth, the SCL-90 captures symptoms experienced within the previous week, reflecting short-term or situational distress rather than enduring psychological adjustment. Temporary stressors—such as adaptation to new teaching responsibilities or workload peaks—may have inflated certain symptom scores. Therefore, the present findings should be interpreted as reflecting recent psychological states rather than stable mental-health conditions. Future research could employ longitudinal or trait-based mental-health measures to examine more persistent patterns.

Finally, the present analysis did not incorporate contextual or workload-related control variables (e.g., field of study, teaching or research intensity, university size, or physical work environment). However, all participants were newly appointed doctoral faculty within three universities that share similar evaluation systems, recruitment criteria, and faculty-support structures, which helped ensure basic comparability of working conditions. Future studies should extend this work by including such contextual and organizational variables to better delineate the influence of workplace factors on the personality–mental-health linkage.

## 5 Conclusion

Young doctoral faculty exhibit moderate, interpretable multivariate associations between personality profiles and mental-health outcomes: approach-oriented, emotionally stable individuals show fewer interpersonal and somatic complaints, whereas tension and apprehension relate to broader symptom loads. The stronger association in men reflects sociocultural expectations and gender-role socialization within academia. Situated within global evidence yet shaped by local institutional norms, these findings underscore the need for person-environment-informed, culturally attuned faculty-support programs that integrate psychological well-being into academic development and policy frameworks.

## Data Availability

The datasets presented in this study can be found in online repositories. The names of the repository/repositories and accession number(s) can be found in the article/supplementary material.

## Footnotes

1
https://www.ibm.com/cn-zh/products/spss-statistics
